# Gegenexpert*innen: Umwelt, Aktivismus und die regionalen Epistemologien des Widerstandes

**DOI:** 10.1007/s00048-022-00350-x

**Published:** 2022-10-17

**Authors:** Nils Güttler

**Affiliations:** Institut für Geschichte, Universität Wien, Wien, Österreich

**Keywords:** Wissenschaftlicher Aktivismus, Umwelt, Umweltwissenschaften, Soziale Bewegungen, Region, Scientific activism, Environment, Environmental sciences, Social movements, Region

## Abstract

Mit der Nachfrage nach „Gegenwissen“ in den sozialen Bewegungen der 1970er und 1980er Jahre wurden „Gegenexpert*innen“ zu einem integralen Bestandteil der Umwelt- und Gesundheitspolitik. Im Umweltbereich waren sie besonders in den ökologisch stark belasteten Industrieregionen und Ballungsgebieten aktiv. Das regionalistische Problembewusstsein der Gegenexpert*innen korrelierte, so die These des Aufsatzes, mit einem Modus von Wissenschaft, der für die Umweltwissenschaften im 20. Jahrhundert insgesamt typisch war. Die Geschichte der Umweltwissenschaften war über das gesamte 20. Jahrhundert hinweg eine Geschichte regionaler Epistemologien. Die Gegenexpert*innen waren aus dem nahräumig formatierten und infrastrukturnahen Feld des Umweltwissens bald nicht mehr wegzudenken. Der Aufsatz rekonstruiert, ausgehend von jeweils einer Publikation, drei umweltpolitische Projekte von Gegenexpert*innen im Rhein-Main-Gebiet um 1980: das Engagement des Umweltpfarrers Kurt Oeser; der epistemische Aktivismus der Gründungsgrünen Jutta Ditfurth; sowie das Programm einer „sozialen Naturwissenschaft“ im Umfeld des Darmstädter Wissenschaftsphilosophen Gernot Böhme.

## Abstract (en)

With the demand for “counter-knowledge” in the social movements of the 1970s and 1980s, “counter-experts” became an integral part of politics. In the field of environmental activism, counter-experts were particularly well represented in regions and agglomerations with high levels of industrial pollution. This essay argues that awareness correlated with a mode of knowledge production that was typical for the environmental sciences in the twentieth century. The history of the environmental sciences throughout that period was shaped by regional epistemologies, often emerging in the context of large-scale infrastructural projects. Many counter-experts therefore had strong ties with the field of the environmental sciences. The article traces three influential counter-experts in the Frankfurt Main region by 1980: the pastor Kurt Oeser; scientific green activist Jutta Ditfurth; and the project of a “social natural science” related to the Darmstadt philosopher Gernot Böhme.

## Problemfall Rhein-Main-Gebiet

„O Jahrhundert, O Wissenschaft – es ist eine Lust Kunst zu leben.“ Auf einer Karikatur, die im Februar 1970 in der *Frankfurter Allgemeinen Zeitung *erschien, war ein Mann zu sehen, der am Rande einer Straße inmitten von Industrie‑, Autoabgasen und einem verseuchten Fluss förmlich den Verstand verlor. In dem dazugehörigen Artikel „Der Mensch als Feind der Natur“ bot der Journalist Rolf-Martin Korda auf, was das „Modethema“ Umwelt – so die Formulierung des Autoren – zu bieten hatte: das globale Bevölkerungswachstum, schmelzende Polkappen, verschmutzte Flüsse, sterbende Wälder, wachsende Müllberge, der ohrenbetäubende Lärm infolge der „Allgegenwart von Autos, Flugzeugen und Maschinen“ (Korda 1970). Die ökologische Misere der Gegenwart verdichtete sich im Rhein-Main-Gebiet in besonderem Maße.^1^ Die Region war nach Ansicht des Journalisten auf dem Weg, dem bisherigen „Sorgenkind der Bundesrepublik“ in Sachen Umwelt, dem Ruhrgebiet, den Rang abzulaufen (Korda 1970).

Das öffentliche Bild von „Rhein-Main“ als zivilisatorischem Problemfall verfestigte sich in diesen Jahren. Im Frankfurter Umland offenbare sich das ganze Spektrum an Umweltproblemen, befand auch Wolfgang Bartsch, Leiter des Wissenschaftsressorts der *Frankfurter Rundschau*, in seiner 1972 erschienen Ökoreportage *Umweltschutz – Menschenschutz*, eine Mischung aus Rachel Carsons *Silent Spring* (1962) und Alexander Mitscherlichs *Die Unwirtlichkeit unserer Städte* (1965). Bartsch identifizierte neben politischer Fehlplanung zwei weitere Gründe, warum sich die ökologische Situation in der Region derart verschärft hatte: die boomende Chemieindustrie am Untermain und das rasante Wachstum von Verkehrsinfrastrukturen, insbesondere am und rund um den Frankfurter Flughafen. Letzterer sorgte schon seit einiger Zeit für Unmut in der Region: „Unsere Gesundheit und unser Lebensraum sind bedroht! […] Fluglärm macht krank!“, war auf einem Flugblatt zu lesen, das die „Interessengemeinschaft zur Bekämpfung des Fluglärms“ im Frühjahr 1965 verteilte (Bartsch 1972: 56).

Angeführt wurde der Widerstand gegen die regionale Umweltverschmutzung, der sich in zahlreichen lokalen und städtischen Initiativen im ganzen Rhein-Main-Gebiet niederschlug (Lieb 2021), über mehrere Jahre von Kurt Oeser, protestantischer Pfarrer in der Anrainergemeinde des Flughafens Mörfelden. Im Zuge seines Engagements, das bis in die Startbahn-West-Bewegung reichte, machte Oeser Karriere innerhalb der Evangelischen Kirche und wurde als „Umweltpfarrer“ deutschlandweit bekannt. Oeser erwarb sich ein Wissen über ökologische Sachverhalte, mit dem er zum Sprecher für die Sorgen der lokalen Bevölkerung aufstieg, gleichzeitig aber auch als Experte in Kommissionen, als Sachverständiger in Gerichtsverfahren und öffentlichen Anhörungen, und schließlich als Lehrbeauftragter an Fachhochschulen und Universitäten in Erscheinung trat. Oeser nahm eine soziale Rolle im Bereich des Wissens ein, die es in dieser Form bis dato so nicht gegeben hatte und die zeitgenössische Beobachter*innen als Begleiteffekt der aufziehenden „Risikogesellschaft“ deuteten (Beck 1986). Dieser Typus steht im Zentrum des folgenden Artikels: der Gegenexperte, und anfangs seltener, bald häufiger: die Gegenexpertin.

Die Gegenexpert*innen – im Folgenden verwende ich, im Unterschied zur damaligen Praxis, die genderneutrale Form – waren zunächst das Produkt der Nachfrage nach „Gegenwissen“ innerhalb der sozialen Protestbewegungen der 1970er Jahre (Stadler et al. 2020). In der Bundesrepublik tauchten sie in breiterer Form in den Auseinandersetzungen um die Notstandsgesetzgebung auf. Danach waren sie wie in vielen westlichen Ländern besonders in der Frauen‑, Gesundheits- und Umweltbewegung aktiv (Nowotny 1982; Michel & Spengler 1986; Rucht 1988; Murphy 2006). Die Gegenexpert*innen wurden zu einem integralen Bestandteil einer neuartigen Wissenschafts- und Technopolitik. Sie schleusten die Sorgen der Bevölkerung, angereichert mit wissenschaftlicher Expertise, in den politischen Diskurs ein und entfalteten auf regionaler Ebene, häufig rund um große Infrastrukturprojekte, eine nachhaltige Wirkung (Thießen 2013). Die Gegenexpert*innen „unterlaufen die Barriere zwischen der vorgeblich interessenneutralen Welt wissenschaftlicher, technischer und juristischer Experten und der vorgeblich „expertenfreien“ Welt des interessengeleiteten Alltags“, beobachtete in den späten 1980er Jahren der Soziologe und Bewegungsforscher Dieter Rucht (Rucht 1988: 291).

Weder die Sozial- und Politikgeschichte der Umweltbewegung noch die Wissenschafts- und Technikgeschichte hat sich seitdem sonderlich um die Gegenexpert*innen gekümmert.^2^ Dies liegt zunächst daran, dass diese sich nicht, wie schon Ruchts Zitat andeutet, in die Unterscheidung von akademischen Laien und wissenschaftlichen Expert*innen fügen. Selbst die jüngere wissenschaftshistorische Forschung konzentriert sich bevorzugt auf jene Expert*innen, die über eine fachwissenschaftliche Ausbildung verfügten, innerhalb etablierter akademischer Strukturen agierten und den Graben zwischen Wissenschaft und Politik nur selten übertraten, etwa indem sie Gutachten schrieben oder bei öffentlichen Anhörungen in Erscheinung traten (Bocking 2004; Hirschi 2018; Oreskes et al. 2019). Viele Gegenexpert*innen lassen sich in dieser akademischen Landschaft kaum verorten. Wenn man so will, bewohnten sie den epistemologisch schmutzigen Graben zwischen Wissenschaft und Politik, den „richtige“ Expert*innen tunlichst vermieden. Während die bisherige Geschichte des wissenschaftlichen Expertentums also wenig Hilfestellung bietet, ist ein produktiverer historiographischer Referenzpunkt die Geschichtsschreibung zu „citizen science“ und Populärwissenschaft, die über ein facettenreicheres analytisches Instrumentarium verfügt, um die Graubereiche zwischen Laien und Expert*innen zu vermessen (Strasser et al. 2019: 60–62).

Ein weiterer Grund, weshalb Gegenexpert*innen in der Wissenschaftsgeschichte unterbelichtet blieben, liegt in der Geographie ihrer Wissensproduktion. Denn ihr Operationsradius war räumlich stark begrenzt. Der deutschlandweite Bekanntheitsgrad von Gegenexpert*innen wie dem Umweltpfarrer Oeser, dem konvertierten Atomlobbyisten Klaus Traube oder dem linksaktivistischen Bremer Physiker Jens Scheer bildete eher die Ausnahme als die Regel. Häufig blieb das Engagement der Gegenexpert*innen mit jenen Orten verknüpft, gegen die sie ihr Wissen mobilisierten, also mit den Atomkraftwerken und -endlagern, Chemiewerken, Flughäfen, Autobahnen oder Giftmüllanlagen. Das Gegenwissen, das sie produzierten, war in diesem Sinne „situiert“.^3^ Innerhalb der Umweltbewegung korrelierte ihr regionalistisches Problembewusstsein mit einem Modus von Wissenschaft, der für die Umweltwissenschaften insgesamt typisch war. Denn die Geschichte der Umweltwissenschaften war über das gesamte 20. Jahrhundert hinweg eine Geschichte regionaler Epistemologien (Güttler 2019). Die Gegenexpert*innen fügten sich in das nahräumig formatierte Feld des Umweltwissens gut ein und waren aus ihm bald nicht mehr wegzudenken.

Eine Beschäftigung mit den Gegenexpert*innen kann also dazu beitragen, unser Bild von der Geschichte der Umweltbewegung zu differenzieren und zu erweitern. Darüber hinaus regt diese Beschäftigung dazu an, die Geschichte der Umweltwissenschaften neu zu denken – im Sinne einer politischen Wissensgeschichte (Roelcke 2010; Espahangizi & Wulz 2020; Güttler 2020; Wulz et al. 2021). Eine solche politische Wissensgeschichte ist eng mit der Geschichte der technischen Umwelten verknüpft, die seit dem 19. Jahrhundert überall in Europa und anderen Teilen der Welt entstanden. Dies ist auch der Grund, warum sich der folgende Artikel auf das Rhein-Main-Gebiet konzentriert. Hier verdichteten sich wie in anderen Industrieregionen Umweltprobleme auf engsten Raum, was wiederum einen „interdisziplinären“ Austausch zwischen verschiedenen wissenschaftlichen Feldern begünstigte. Gleichzeitig vernetzte sich dieses Wissen, katalysiert durch boomende Verkehrs- und Informationsinfrastrukturen wie dem Flughafen, nach außen. Die Rolle der Gegenexpert*innen bestand darin, auf regionaler und transregionaler Ebene den interdisziplinären Austausch in Umweltfragen zu moderieren, und den politischen Druck zu erhöhen, um „Umwelt“ auf die Agenda der Wissenschaftsförderung zu setzen.

Der folgende Aufsatz rekonstruiert, ausgehend von jeweils einer Publikation, drei umweltpolitische Projekte von Gegenexpert*innen im Rhein-Main-Gebiet zwischen Mitte der 1960er und den späten 1980er Jahren. Es werden unterschiedliche soziale Figuren von Gegenexpert*innen sichtbar und damit auch unterschiedliche gesellschaftspolitische Facetten des ökologischen Gegenwissens. Trotz aller lokalen Spezifik und falltypischen Besonderheit können sie als stellvertretend für das heterogene Milieu der Produktion von Gegenexpertise innerhalb der Umweltbewegung betrachtet werden: das Engagement des erwähnten Umweltpfarrers Oeser; der epistemische Aktivismus der Gründungsgrünen Jutta Ditfurth; sowie das Programm einer „sozialen Naturwissenschaft“ im Umfeld des Darmstädter Wissenschaftsphilosophen Gernot Böhme. Alle drei Projekte drehen sich auf die eine oder andere Weise um eine Ressource, die auch für das Rhein-Main-Gebiet namensgebend war: das Wasser.

## Dialog und Teamwork

„Wasser ist eine, wenn nicht sogar *die *entscheidende Lebensgrundlage für Pflanzen, Tier und Mensch“ (Oeser 1982: 5). Mit diesen Worten begann Umweltpfarrer Oeser das Vorwort zu dem Band *Wasser: Wie ein Element verschmutzt und verschwendet wird *aus dem Mai 1982. Der Band erschien in der Reihe *fischer alternativ*, in der sich das ganze Spektrum „alternativer“ Wissensproduktion bündelte. Der Band selbst konzentrierte sich auf den Bereich der „ökologisch orientierten“ und wissenschaftlich fundierten „Wasserpolitik“ (so der Titel eines Aufsatzes). Zu diesem Zweck waren verschiedene Expert*innen aus den Bereichen der Limnologie und Ökologie mit Praktiker*innen aus dem Recht, der staatlichen Verwaltung und dem Umweltschutz in Austausch getreten. In seinem Vorwort kombinierte Oeser verschiedene Genres: überblicksartige Beschreibungen der gegenwärtigen ökologischen Situation, Zahlen und Statistiken, Zusammenfassungen von wissenschaftlichen Studien und policy-papers sowie, eher beiläufig, schöpfungstheologische Versatzstücke. Letztere kamen immer dann ins Spiel, wenn es darum ging, ethische Schlüsse aus der wissenschaftlichen Forschung zu ziehen (Oeser 1982: 6).

Der Wasser-Band gibt wichtige Hinweise auf das Denkkollektiv des Umweltpfarrers. Oesers Mitherausgeber waren keine Theologen, sondern hatten einen dezidiert fachwissenschaftlichen Hintergrund. Hartmut Bossel war Professor für Umweltschutz an der Gesamthochschule Kassel (Schwerpunkt: Systemforschung); Hans-Joachim Grommelts arbeitete als wissenschaftlicher Mitarbeiter ebenfalls in Kassel und beschäftigte sich mit Landwirtschaft und Ökochemie. Die ungewöhnliche Kollaboration kam dadurch zustande, dass alle drei Autoren dem Wissenschaftlichen Beirat des Umweltbeauftragten der Evangelischen Kirche Deutschland angehörten, den Oeser selbst ins Leben gerufen hatte. In diesem „interdisziplinär zusammengesetzten“ Gremium teilten die Fachwissenschaftler*innen ihre „Detailkenntnisse aus verschiedenen Disziplinen“, wie Oeser schrieb, zum Teil in recht „kontroversen“ Gesprächen (Oeser 1982: 7). Das Gremium sollte der Kirche helfen, zu einem so „schwierigen“ und „komplexen“ gesellschaftspolitischen Thema wie Wasser Stellung zu beziehen (Oeser 1982: 7; vgl. Schüring 2015: 172–233). Es brachte gleichzeitig Personen aus den Naturwissenschaften, Verwaltung und Politik miteinander in Kontakt. Dabei zeigte sich, dass Oeser eine spezifische Expertise des Pfarrberufs in die umweltwissenschaftliche Diskussion mit einbrachte: den Dialog. Der Dialog wiederum war eine Voraussetzung für den „interdisziplinären“ Zugang zur Umwelt, den der Band anstrebte, mit dem sich aber viele Fachwissenschaftler*innen schwertaten.^4^

Man versteht die soziale und diskursive Position Oesers im Bereich des Umweltwissens besser, wenn man an den Beginn seiner Aktivität als Gegenexperte zurückblickt. Sein ökologisches Bewusstsein bildete sich ursprünglich nicht am Wasser, sondern an der unangenehmsten konkreten Begleiterscheinung des Düsenflugzeitalters heraus: dem Fluglärm. Oeser war schon früh in der Anti-Fluglärm-Bewegung im Umfeld des Frankfurter Flughafens aktiv. Im November 1964 prangerte er in einem offenen Brief an den Hessischen Ministerpräsidenten die unerträgliche „Situation im Ballungsraum Rhein-Main“ an und trat damit eine „Lawine“ der Entrüstung in der Region los (Bartsch 1972: 52–55, Zitat 55). Nach der Gründung der ersten örtlichen „Interessengemeinschaft zur Bekämpfung des Fluglärms“ engagierte sich Oeser für einen deutschlandweiten Zusammenschluss der lokalen aktivistischen Gruppen. Die „Bundesvereinigung gegen Fluglärm“ wurde im November 1967 mitten in der Einflugschneise des Frankfurter Flughafens, in Neu-Isenburg, gegründet (Oeser 1978b). Noch im gleichen Jahr begann der westdeutsche Verband Kontakt mit Anti-Lärm-Kampagnen in ganz Europa aufzunehmen, zunächst mit Schwerpunkten in der Schweiz und Frankreich (Oeser 1978b: 8).^5^ Im Folgejahr entstand die europäische Anti-Fluglärm-Vereinigung (Oeser 1969: 10–13). Die rasche Europäisierung und Internationalisierung der Bewegung entsprach der Logik des Fluglärms. Aus Perspektive der Aktivist*innen war der Austausch zwischen den lärmbelasteten Flughafenregionen Europas – Rhein-Main, Zürich, Amsterdam, der Pariser und Londoner Agglomeration – die logischere Vernetzungsebene als eine rein nationale Interessenvertretung.

Die Anti-Lärm-Initiativen verfolgten zwei miteinander verschaltete Strategien. Nach außen übten sie Druck auf die Entscheidungsträger*innen in Politik und Wirtschaft aus. Ein erster Erfolg war im westdeutschen Fall die Verabschiedung eines bundesweiten Fluglärmgesetzes im März 1971, in dem unter anderem die Einrichtung von Lärmschutzzonen verankert wurde;^6^ kurz darauf gab die Deutsche Forschungsgemeinschaft (DFG) eine große Fluglärmstudie in Auftrag (Rohrmann 1974), auf der später eine Reihe von Verordnungen und Durchführungsmaßnahmen aufbauten. Nach innen verstanden sich die Anti-Fluglärm-Initiativen als Plattformen, um „anderes“ Wissen in Sachen Fluglärm zu produzieren und möglichst weit zu streuen – meist in Form von Kompendien und Informationsbroschüren. Oeser setzte sich schon früh für die Gründung eines wissenschaftlichen Beirats ein, der die Bundesvereinigung unterstützten sollte. In Arbeitsgruppen widmeten sich „Fachleute“ spezifischen Aspekten der Lärmproblematik: Recht und Verwaltung, Medizin, Pädagogik, Psychologie, Geologie, Biologie, Physik, Chemie, Flugnavigation und -technik (Bartsch 1972: 58–59). Das Problem Fluglärm forcierte damit ähnliche Kollaboration zwischen Wissenschaft und Bürgerinitiativen, wie sie seit den 1950er Jahren im Bereich des Verbraucherschutzes entstanden waren (Stoff 2015). Die Bundesvereinigung sah ihre Aufgabe darin, dieses Wissen möglichst breit zurück in die örtlichen Initiativen zu leiten.

Die Wissensproduktion war aus Sicht der Fluglärmgegner umso dringlicher, als sich die Luftfahrtindustrie längst wissenschaftliche Schützenhilfe besorgt hatte. Die Anti-Lärm-Initiativen beklagten sich wiederholt über die „Manipulation“ (Oeser 1974: 25) der öffentlichen Meinung durch Flughäfen und Luftfahrtindustrie, etwa durch tendenziöse Gutachten und Medienkampagnen. Oeser & Co. stellten schnell fest, dass die „mächtige Lobby“ (Oeser 1978b: 7) aus Industrie und Politik mit jenem Bereich der Lärmforschung auf besonders gutem Fuß stand, der sich auf objektivierbare Messverfahren – wie die Messung des Schalldruckpegels in Dezibel – konzentrierte.^7^ Das wichtigste Pro-Flughafen-Lärmgutachten dieser Zeit stammte von dem Arbeitsmediziner Gerd Jansen, Mitarbeiter am einflussreichen Max-Planck-Institut für Arbeitsphysiologie in Dortmund (später wurde er Leiter des Instituts für Arbeitsmedizin an der Heinrich-Heine-Universität Düsseldorf). Im Jahr 1968 attestierte Jansen dem Flughafen eine teilweise positive Lärmwirkung (Jansen 1968: 93). Die Bürgerinitiativen liefen Sturm. Noch rund ein Jahrzehnt später, im großen Landtagshearing zur Startbahn West im Februar 1981, warfen sie dem Arbeitsmediziner vor, er habe seine Daten „manipuliert“ und schöngerechnet (Hessischer Landtag 1981: 245).

Als Reaktion nahm die deutsche Anti-Fluglärm-Initiative rund um Oeser schon früh Kontakt zu Wissenschaftler*innen auf, die ein „sozialeres“ Konzept von Lärm vertraten und boten ihnen eine öffentliche Bühne. Eine der wichtigsten Ansprechpersonen war der Arbeitsphysiologe Gunter Lehmann. Lehmann leitete das Max-Planck-Institut für Arbeitsphysiologie in Dortmund (wo auch der erwähnte Jansen tätig war) und war seit den 1950er Jahren Vorsitzender des Arbeitsrings für Lärmbekämpfung. Lehmann öffnete die von ihm mitherausgegebene Zeitschrift *Kampf dem Lärm *für Gegenexpert*innen wie Oeser. Ein anderer wichtiger Kontakt für die Bewegung wirkte an der Eidgenössischen Technischen Hochschule (ETH) in Zürich. Hier hatte der Arbeitsphysiologe und Hygieniker Etienne Grandjean damit begonnen, seine Forschungen zur „Umwelt“ von Arbeitsplätzen in der Fabrik oder im Büro verstärkt in den Außenbereich – also unter freien Himmel – zu verlegen (Kryter & Grandjean 1960). In der Wissenstradition der Arbeitshygiene (vgl. Sellers 1997) war Lärmbekämpfung nicht mehr auf die Regulierung der Lärmquelle reduzierbar, sondern wurde zu einer komplexen gesellschaftlichen Aufgabe, in die etwa auch die Regionalplanung und Raumordnung miteinbezogen werden musste (Grandjean & Gilgen 1973). Es etablierten sich auf diesem Wege Schnittstellen zwischen der Arbeitsphysiologie und anderen Wissensgebieten wie der Rechts- und Verwaltungswissenschaft, der Biologie und Chemie, aber auch der Soziologie und Psychologie.

Oeser sah seine Aufgabe darin, diese Forschungsgebiete miteinander in Austausch zu bringen. „Ohne ständigen Dialog kommen wir nicht weiter“ (Oeser 1969: 14). Dieser Dialog fand seinen Niederschlag in Arbeitsgruppen innerhalb der Bundesvereinigung gegen Fluglärm. Die Aktivität der Wissenschaftler*innen im Umfeld der Bürgerinitiativen zeichnete sich, so Oesers Einschätzung, durch zwei Merkmale aus: „Teamwork“ sowie eine gemeinsame ethische Haltung, die der Umweltpfarrer als „‚Bruderschaft im Sachlichen‘ um der Menschen Willen“ charakterisierte (zitiert nach: Bartsch 1972: 59). Ob sich in dieser Beschreibung alle beteiligten Wissenschaftler*innen wiederfanden, sei dahingestellt. Sicher ist, dass das gemeinsame gesellschaftspolitische Ziel der Reduktion des Fluglärms tatsächlich viele Fachwissenschaftler*innen zwang, öffentlich Stellung zu beziehen.^8^ Viele industriekritische Studien, die in den folgenden Jahren im Umfeld der Bundesvereinigung gegen Fluglärm entstanden, dienten als Beleg vor Gericht (vgl. etwa: Anonym 1972). Das öffentlich aufgeheizte Thema Fluglärm generierte eine Aufmerksamkeit für die „kritischen“ Wissenschaftler*innen in den Medien, die sie anderweitig kaum erhalten hätten (Löbsack 1967; Anonym 1964). Oeser gelang es sogar, namhafte DFG-Gutachter*innen, wie etwa den Psychologen Bernd Rohrmann, in seine Informationskampagnen einzuspannen (Oeser 1978a).

Die politische Aktivität der Lärmforscher*innen katalysierte eine „interdisziplinäre“ Perspektive auf Lärm. „Die Bundesvereinigung“, erklärte Oeser, „hält mehr interdisziplinäre Forschungsarbeiten für unumgänglich und bedauert, daß bisher […] durchweg noch zu stark Einzelforschung betrieben wird“ (Oeser 1978b: 9). Die ersten Resultate dieser Kooperationen ließen sich im März 1969 in Wiesbaden beobachten. Auf Einladung des Deutschen Arbeitsrings für Lärmbekämpfung und der Bundesvereinigung gegen Fluglärm versammelten sich rund 350 Expert*innen in der Hessischen Landeshauptstadt zum Thema Fluglärm. Im Plenum befanden sich neben Vertreter*innen aus dem Bereich der Akustik, Medizin, Arbeitsphysiologie, Psychologie, Raumplanung und Physik auch Entscheidungsträger*innen aus Politik und Wirtschaft wie etwa die damalige Bundesgesundheitsministerin Käte Strobel. Hinzu kamen Abgesandte der Flughäfen, der Flugsicherung und Airlines sowie Mitglieder des Bundestages (Graßmann 1969). Zwei Jahre später fand in Zürich der „Internationale Kongreß Fluglärmbekämpfung“ statt, dessen wissenschaftliches Programm stark vom ETH-Arbeitsphysiologen Grandjean geprägt wurde.^9^ Oeser trat bei solchen Kongressen als Redner in Erscheinung und verkörperte damit die schleichende Vermischung von sachlicher Expertise und politischem Engagement.

Für all diese Aktivitäten erwies sich Oesers kirchlicher Hintergrund von Vorteil. Nicht nur brachte seine Stellung als Pfarrer genügend gesellschaftliche Autorität mit sich, um auch als Laie zu kontroversen wissenschaftlichen Themen Stellung zu beziehen. Darüber hinaus nutzte Oeser die institutionellen Strukturen der Kirche, um Umweltthemen eine breitere gesellschaftliche Plattform zu geben und innerkirchlich Gleichgesinnte zu rekrutieren (Dannemann & Dannemann 1982: 48–49). Innerhalb der Landeskirche Hessen-Nassau etablierte Oeser im Jahr 1970 einen „Gesprächskreis Naturwissenschaft – Landschaft – Mensch“, in dem die ökologischen Sorgen in den Gemeinden im Zentrum standen.^10^ Das regionale theologisch-ökologische Engagement Oesers bildete das Sprungbrett für seine rasante Karriere innerhalb der Evangelischen Kirche Deutschlands (EKD). Und durch diese Karriere widmete er sich bald auch anderen Umweltthemen wie dem Wasser.

Oeser wurde bald darauf zum „Umweltbeauftragten“ der EKD ernannt und koordinierte fortan das Netz an Umweltbeauftragten in den Landeskirchen. Die Kirche trat über die Themen Umwelt und Umweltbewusstsein insgesamt verstärkt als Akteurin im Bereich der Wissenschaftskommunikation auf, insbesondere in der Anti-Atomkraft-Bewegung (Schüring 2015). Sie stellte den Protestbewegungen ihre bestehende Infrastruktur aus Tagungszentren und Bildungseinrichtungen zur Verfügung – die Evangelische Akademie Loccum tat sich hier besonders hervor –, bezog zu tagesaktuellen Themen Stellung oder positionierte sich als Vermittler zwischen Politik und betroffener Bevölkerung. In Reihen wie *fischer alternativ *erschienen deshalb auch theologische Titel wie der Band *Franziskus in Gorleben: Protest für die Schöpfung* (Bahr et al. 1981). Die Umweltbeauftragten wiederum organisierten wissenschaftliche Tagungen oder engagierten sich in der Durchführung von Ausstellungen, etwa des Kassenschlagers „Die Welt, ein vernetztes System“, mit der der Systemökologe Frederic Vester von Stadt zu Stadt zog (Vester 1983; Barner et al. 1986: 12; zu Vester: Kuchenbuch 2016).

Oeser konnte sich über die Jahre als vermittelnde Instanz im Bereich des Umweltwissens positionieren. Allerdings wurde im Rhein-Main-Gebiet der Dialog mit der „Gegenseite“ immer schwieriger, da sich die politischen Fronten durch die Startbahn-West-Auseinandersetzungen verhärteten. Bei all seinem politischen Engagement gerät schnell in Vergessenheit, dass Oeser seinem eigentlichen Beruf beziehungsweise seiner Berufung des Pfarrdienstes weiterhin nachging. Im Hüttendorf, das zur Verhinderung der Flughafenerweiterung im Startbahn-Wald errichtet wurde, predigte er regelmäßig in der „Hüttenkirche“. In seiner täglichen Arbeit durchkreuzte sich Weltliches und Geistliches, Aktivismus und Predigt, Umweltbewusstsein und Schöpfungsglaube, Gegenwissen und Kirche – und befruchteten sich gegenseitig. Oeser bezeichnete seine Debattenbeiträge einmal ganz treffend als „säkulare Predigt“ (Bartsch 1972: 60), also als eine Art des Vortrags, der sich aus den moralischen Ökonomien der Kirche speiste, der aber auf solidem empirischem Fundament stand. Selbst im anti-kirchlich geprägten Alternativmilieu traf seine säkulare Predigt auf Zuspruch. Es mag auf den ersten Blick so erscheinen, als wurde Oeser zu einem Gegenexperten, *obwohl *er evangelischer Pfarrer war. Bei näherem Hinsehen war das Gegenteil der Fall. Oeser wurde auch deshalb Gegenexperte, *weil *er evangelischer Pfarrer war.

## Aktion

„Manche Menschen leben an einem Fluß und kennen ihn kaum“, schrieben die Grünen-Politikerinnen und Umweltaktivistinnen Jutta Ditfurth und Rose(marie) Glaser im Vorwort zu einem anderen Wasser-Buch aus den 1980er Jahren, das bereits im Titel einen deutlich kämpferischeren Ton anschlug: *Die tägliche legale Verseuchung unserer Flüsse und wie wir uns dagegen wehren können. *Den Herausgeberinnen ging es in ihrem *Handbuch mit Aktionsteil *– so der Untertitel – nicht allein darum, auf den „kranken Zustand unserer Flüsse“ hinzuweisen, „ihren Zustand zu beschreiben und traurig oder wütend zur Kenntnis zu nehmen“ (Ditfurth & Glaser 1987). Das Wissen um die Flüsse sollte vielmehr politische Handlungen in der Bevölkerung anstoßen, zu denen staatliche Stellen nicht imstande seien: „Menschen müssen ihre Flüsse selber retten. Industrie, Regierungen und Wachstumsparteien werden es nicht tun. Wir wollen zum Handeln anstiften, ohne die Einmischung von vielen aktiven, informierten und phantasievollen Menschen werden unsere Flüsse zu schlammigen Giftkanälen, trüben Giftmülldeponien, allenfalls Wasserautobahnen und unser Trinkwasser wird zur Giftbrühe“ (Ditfurth & Glaser 1987). Glaser und Ditfurth wurden im Zuge ihrer Recherche selbst zu Wasserexpert*innen und fügten damit dem intellektuellen Profil der Gegenexpert*innen drei wichtige Eigenschaften hinzu: Phantasie, die Bereitschaft, die Produktion wissenschaftlicher Fakten selbst in die Hand zu nehmen, sowie eine „ökosozialistische“ Interpretation des Verhältnisses von Wissenschaft und Gesellschaft.

Das Ergebnis war ein rund 450 Seiten umfassender Band, dessen erster Teil über den Zustand verschiedener Flüsse in Mitteleuropa berichtete, eingeleitet durch den Text „Was ist ein Fluß“ aus der Feder einer gewichtigen öffentlichen Autorität in Sachen Wissenschaft: Hoimar von Ditfurth, Psychiater, Neurologe, Fernsehmoderator und der wohl bekannteste deutschsprachige Wissenschaftspopularisierer seiner Zeit (und Jutta Ditfurths Vater) (von Ditfurth 1987). Der zweite Teil des Handbuchs, der „Aktionsteil“, diente als Nachschlagewerk, in dem von der lokalen bis zur transnationalen Ebene wichtige Ansprechpartner*innen aufgelistet wurden: Umweltinitiativen, Naturschutzverbände und Aktivist*innengruppen, Verbraucherverbände, grüne Parteien, Ministerien und Behörden, „Umwelt(desinformations)telefone“, Datenbanken und andere Informationssysteme. Außerdem fanden die Leser*innen ausführliche Literaturhinweise – von Anleitungen zu Schnelltests bis hin zu Wasserrundbriefen – sowie das ganze Spektrum nichtschriftlicher Medien: Filme, Videofilme, Diaserien, Tonbildschauen und Foliensätze. Das Handbuch gibt ein umfassendes Zeugnis für die immense Produktion und Sammlung von offiziellem und alternativem Wasser- und Umweltwissen, das seit den frühen 1970er Jahren von Dutzenden lokalen und städtischen Umweltgruppen produziert worden war (vgl. für das Rhein-Main-Gebiet: Lieb 2021).

Dass sich die Herausgeberinnen für Flüsse interessierten, hatte nicht nur mit der realen Umweltproblematik, sondern auch mit ihrem politischen Wirkungskreis zu tun. Rose Glaser war bei den baden-württembergischen Grünen aktiv und stand kurz davor, für den Freiburger Wahlkreis ins Landesparlament einzuziehen. Im Breisgau stand zu diesem Zeitpunkt das Brandunglück im Basler Industriegebiet Schweizerhalle vom November 1986, durch das Unmengen an Chemikalien in den Rhein gelangt waren, im Zentrum der öffentlichen Debatte (Deutsche Kommission zur Reinhaltung des Rheins, Arbeitsausschuss 1986). Jutta Ditfurths politisches Engagement im Rhein-Main-Gebiet richtete sich verstärkt gegen die Chemieindustrie am Main und speziell gegen das ehemalige IG Farben-Unternehmen Hoechst. Mit dem Chemieunternehmen war wiederum ein ganzes Netz angelagerter Industrien verbunden. Aufgrund der ungeheuren Nachfrage nach Erdölprodukten hatte sich am Untermain seit den frühen 1960er Jahren die Mineralölindustrie breitgemacht. Im Sommer 1964 eröffnete die US-amerikanische Mineralölgesellschaft Caltex in Raunheim die erste Raffinerie Hessens, in der es wiederholt zu Unglücken kam und Benzine und Chemikalien in den Main gelangten (Anonym 1966). Die Gegenexpert*innen suchten sich also starke Gegner.

Bei einem Blick auf die Landkarte zeigt sich, dass die Orte, an denen sich im Rhein-Main-Gebiet das Gegenwissen entzündete, nah beieinanderlagen. Von Hoechst bis zum Flughafen waren es nur wenige Kilometer und von dort aus war es auch nur ein Katzensprung zum Startbahn-West-Wald und zu Oesers Gemeinde Mörfelden. Der Flughafen wiederum beschäftigte die Anwohner*innen in den 1970er Jahren längst nicht mehr nur als Lärmverursacher. Mit der geplanten Abholzung von rund 300 Hektar Wald für den Bau der Startbahn 18 West drohte der Verlust wichtiger Habitate für Tiere und Pflanzen und die Zerstörung eines wichtigen Naherholungsgebiets für die Bevölkerung. Umweltaktist*innen und Anwohner*innen befürchteten zudem, dass mit der Rodung der regionale Grundwasserhaushalt nachhaltig verändert werden würde. Im Flughafenwald befanden sich große Grundwasserreservoirs, von denen die Trinkwasserversorgung Frankfurts abhing (Gonnermann 1981). Verschiedene Umweltproblematiken verdichteten sich also auf engstem Raum. Für den politischen Umgang mit diesen Problemen mobilisierte die Umweltbewegung auf unterschiedlichen Ebenen Wissen: von der Botanik, Zoologie und Forstwissenschaft, über die Geologie, Hydrologie und Meteorologie, bis hin zur Medizin und Soziologie.^11^

In Sachen Wasser fand der für die Main-Region wissensgeschichtlich entscheidende Vorfall im Winter 1980/81 statt. Nachdem es seit den 1960er Jahren immer wieder zu kleineren und größeren Tank- und Kerosinunfällen auf dem Flughafengelände gekommen war, traten auf dem Höhepunkt der Startbahn-West-Proteste über Monate unbemerkt mehrere Millionen Liter Kerosin aus dem maroden unterirdischen Tankleitungssystem des Flughafens aus und sickerten in den Boden. Als der Vorfall bekannt wurde, empörten sich die Umweltverbände nicht nur über die realen Schäden für Mensch und Natur, sie beklagten auch „widersprüchliche Informationen“ (Anonym 1981f) seitens der Behörden. Der wenige Jahre zuvor gegründete Bund für Umwelt und Naturschutz Deutschland (BUND) sprach gar von „Vertuschungspolitik“ (Anonym 1981e), witterte ein Komplott aus Politik, Wirtschaft und Verwaltung und forderte den Rücktritt des hessischen Umweltministers Karl Schneider (SPD) (Anonym 1981a).

Die Bürgerinitiative gegen den Bau der Startbahn West hielt deshalb nach Expert*innen aus Biologie, Ökologie, Hydrologie, Geologie, Chemie und Physik Ausschau. Die Umweltwissenschaften sollten helfen, den sich anbahnenden Skandal aufzudecken. „[W]er kennt solche Leute?“, hieß es in einem Rundbrief aus dem August 1981.^12^ Die Frankfurter Grünen rund um Ditfurth begannen daraufhin, eigene Messungen in Brunnen in der Umgebung des Flughafens durchzuführen. Weil sie dort Rückstände von Kerosin fanden, gingen sie davon aus, dass die Behörden „geblufft“ und die Sachverhalte „verniedlicht“ hätten (Anonym 1981d).^13^ „Unabhängige Wissenschaftler müßten“, so die Position der Grünen, „das gesamte Trinkwassergewinnungsgebiet in der Flughafennachbarschaft auf Schadstoffe untersuchen“. Am Flughafen und der Umgebung bahne sich „eine Katastrophe“ an (Anonym 1981d).

Im Kerosinskandal wurde ein wichtiges Merkmal der Wissensproduktion von Gegenexpert*innen wie Ditfurth sichtbar: Wissenschaft musste in bestimmten Situationen, in denen offizielle Stellen scheinbar versagten, selbst in die Hand genommen werden. Eine wichtige Voraussetzung hierfür war die Beschaffung von Messinstrumenten. Im Bereich des Lärms waren zu dieser Zeit bereits portable und kostengünstige Lärmmessgeräte beliebt, mit denen die offiziellen Angaben überprüft werden konnten. Bei Schadstoffmessungen im Wasser war der Nachweis komplizierter, da eine Laborauswertung notwendig war. Die Aktivist*innen versuchten, Kräfte zu bündeln. So war beispielweise in der ersten Ausgabe des *umweltexpress *vom August 1981, dem Organ der Bürgerinitiative „Keine Startbahn West“, zu lesen: „Das internationale Komitee Zum Schutz Des Rheins [sic] hat ein 16 m langes Boot gekauft, das, als Laborboot umgebaut, Wasserproben aus Rhein und Main entnehmen soll. Auf einer internationalen Konferenz wurde beschlossen, daß die Jungfernfahrt des Bootes eine Fahrt gegen die Startbahn West und für das Volksbegehren sein soll“ (Anonym 1981b). Andernorts entstanden unabhängige „Umweltlabors“. Das erste dieser Art im Rhein-Main-Gebiet wurde von der Arbeiterhilfe Oberursel ins Leben gerufen. Hier sollten „aktive Bürger und Gruppen“ nach kurzer Einarbeitung an den Maschinen die Möglichkeit erhalten, Umweltschäden selbst festzustellen, Schadstoffe direkt zu messen und biologische Produkte zu kontrollieren (Anonym 1983).

Die Aktivitäten richteten sich bald schon nicht mehr gegen den Flughafen allein, sondern zogen größere Kreise. Um beispielsweise die nachhaltige Schädigung von Arbeiter*innen und die Verseuchung von Fluss und Boden durch Amine und andere krebserregende Stoffe nachzuweisen, entnahm Ditfurth mit anderen Aktivist*innen im Juni 1985 Boden- und Wasserproben vom Hoechst-Werksgelände, dem sie sich mit Schlauchbooten genähert hatten (Ditfurth 1988). Ditfurths Aktivismus umfasste in diesen Jahren aber nicht nur Messungen – samt ihrer medialen Inszenierung –, sondern auch die Produktion von historischem Gegenwissen. In dem Wasser-Handbuch war sie mit einem Beitrag vertreten, der die nationalsozialistische Vergangenheit von Hoechst und anderer Chemieunternehmen thematisierte (Ditfurth & Zieran 1988; vgl. Lindner 2005). Bei offiziellen Anlässen wie der BASF-Hauptversammlung versuchte sie, dieses Wissen gegen den Chemiekonzern in Stellung zu bringen (Ankenbauer-Grüber 1989).

Wie das Gegenwissen konkret in den politischen Prozess eingespeist wurde, lässt sich anhand einer Szene aus dem Frankfurter Stadtparlament illustrieren. Im Mai 1981 stand die örtliche Grundwassersituation zur Diskussion. Das wenige Jahre zuvor gegründete Freiburger Öko-Institut hatte eine groß angelegte Studie vorgelegt: „Bei der Diskussion über die Tagesordnung stellte Jutta Ditfurth den anderen Abgeordneten die Frage, ob sie interessiert seien, die neuesten Untersuchungsergebnisse über das Frankfurter Trinkwasser […] zu erfahren. Die CDU lehnte dies gleich ab, die SPD beantragte eine Unterbrechung der Sitzung. Die SPD-Abgeordneten zogen sich zu einer schnellen Fraktionssitzung an der Saaltür zurück, um sich zu beraten. Sie befanden sich in der Zwickmühle, denn inhaltlich waren sie schon an den neuesten Informationen interessiert, wollten aber andererseits ihren eigenen Mann in der Frankfurter Verwaltung decken“ (Lahl & Zeschmar-Lahl 1984: 142).

An diesem Punkt unterschied sich die jüngere Generation deutlich von den Gegenexpert*innen älteren Schlages, wie ihn Oeser vertrat. Setzte der Umweltpfarrer darauf, die bestehenden Strukturen im Bereich des Wissens für eigene Zwecke zu nutzen und im Dialog zu verändern – Oeser war beispielsweise jahrelanges Mitglied der offiziellen und industrie- und staatsnahen „Kommission zur Abwehr des Fluglärms“ –, misstrauten Ditfurth & Co. genau jenen Strukturen. Stattdessen versuchten sie, ihre Informationen aus politisch „verlässlicheren“ Quellen zu erhalten, die in dem *Handbuch mit Aktionsteil *detailliert aufgelistet wurden: lokale Umweltinitiativen, unabhängige Forscher*innen oder alternative Forschungsinstitute, die sich wie das Freiburger Öko-Institut um 1980 außerhalb der Universitäten etablierten. Diese Strategie wurde mit einer Menge Ideologiekritik unterfüttert, die Oeser ebenfalls fremd gewesen wäre. Ditfurth übertrug die politischen Strategien der Studentenbewegung – Rudi Dutschke gehörte in den 1980er Jahren zu einer häufigen Referenz in ihren Schriften – auf den Bereich der Naturwissenschaften, für den sich „1968“ im Detail noch wenige interessiert hatten. Auch hier könne man die Produktionsmittel selbst in die Hand zu nehmen. „Authentisches“ und „selbstbestimmtes“ Leben war selbst im Bereich des Wissens möglich.^14^

So anti-bürgerlich sich Ditfurth & Co. auch gaben, die Idee, Wissenschaft selber zu machen, hatte eine lange bürgerliche Tradition und reichte bis in die populäre Wissenschaftskultur des 19. Jahrhunderts zurück. Im Rhein-Main-Gebiet erlebte diese Art von populärer Naturkunde, wie die Wissenschaftshistorikerin Ayako Sakurai gezeigt hat, bereits in den 1880er und 1890er Jahren einen ersten Höhepunkt, als Naturforscher*innen aus dem Umfeld der Senckenbergischen Gesellschaft das Frankfurter Umland für sich entdeckten (Sakurai 2013: 119–146). Im Umfeld dieser populärwissenschaftlichen „Heimatforschung“ vor dem Ersten Weltkrieg wurden auch Versuche unternommen, die Naturgeschichte soziopolitisch zu mobilisieren, indem Arbeiter*innen der Fabriken am Main in die naturkundlichen Sammlungsprojekte integriert wurden (Kobelt 1912: 119). Es gab in der Region also durchaus eine bürgerliche Tradition für die Mobilisierung des Umweltwissens „von unten“.

Zweifellos unterschied sich die Situation um 1980 dennoch in vielen Punkten von der paternalistischen Naturkunde des Kaiserreichs. Zunächst hatte sich die Zahl potenzieller Gegenexpert*innen infolge des Bildungsbooms der 1960er und 1970er Jahre erweitert. Ein Beispiel hierfür ist Rose Glaser, Mitherausgeberin des Wasser-Handbuchs, die auf dem zweiten Bildungsweg eine Ausbildung zur Grund- und Hauptschullehrerin absolvierte (Hochreuther 2012). Die vielleicht wichtigste Veränderung vollzog sich aber zweifellos in den wandelnden Geschlechterrollen. War die Populärwissenschaft des 19. Jahrhunderts weiterhin männerdominiert gewesen, strömten um 1980 verstärkt Frauen in den Bereich der alternativen Wissensproduktion. Als Gegenexpertinnen aufzutreten, bot Glaser und Ditfurth die Möglichkeit, auch als „Amateurinnen“ im Bereich des Wissens mitzuspielen.

## Soziale Naturwissenschaft

Zum Ende des Wintersemesters 1982/83 erschien als Abschluss eines fächerübergreifenden Projektseminars an der Technischen Hochschule Darmstadt die *Wasser-Zeitung*, eine Sammlung von schriftlichen und bildlichen Materialien zur Wissens‑, Kulturgeschichte und Ökologie des Wassers. Für den Seminarleiter Gernot Böhme, Technikphilosoph an der TH Darmstadt, wurden in der Zusammenstellung unterschiedliche soziale und gesellschaftliche Umgangsweisen mit „Natur“ sichtbar: „Für eine Gruppe ist z. B. ein Fluß ein Transportweg für Güter, für andere dient er zum Abtransport von Schadstoffen, ist Trinkwasserreservoir, Medium für die Aufzucht von Nutztieren oder dient als Erholungsort“ (Deneke 1983: 1). Die an der *Wasser-Zeitung *beteiligten Studierenden schlugen deutlich schärfere Töne an. Der einleitende Bericht zur Exkursion entlang des durch Darmstadt fließenden Darmbachs, der flussabwärts in „Europas Kloake“, den Rhein, mündete, bestand hauptsächlich aus einer Aufzählung von Verunreinigungen (Deneke 1983).

Auch in dem Landstrich zwischen Darmstadt und dem Rhein, dem Hessischen Ried, sah es kaum besser aus (Deneke 1983: 80–82). In dem ehemals sumpfigen Überschwemmungsgebiet des Rheins, das sich vom Rhein-Main-Gebiet südlich erstreckte, hatten sich mehrere Industrieunternehmen und Wasserverbände angesiedelt, die hier – in den Worten von Mitarbeiter*innen des Freiburger Öko-Instituts – „um die Wette pumpten“ (Lahl & Zeschmar-Lahl 1984: 79). Während immer neue Brunnenanlagen den steigenden Trinkwasserbedarf des Ballungsraums Frankfurt sichern sollten, sank vor Ort der Grundwasserspiegel. Das Hessische Ried hatte um 1980 überregionale Berühmtheit erlangt und die örtliche Wasserkrise komplettierte ein düsteres Bild des schonungslosen Umgangs mit Wasser in ganz Deutschland (Kübler 1981: bes. 93–108). So stellte sich auch für die Darmstädter Studierenden die Frage, ob die Trinkwassergewinnung im Ried „derart forciert werden muß“: „Es wird hier ein Gebiet extrem zur Wasserversorgung belastet – und das nicht erst seit heute! Davon profitiert ein Ballungsraum, der nicht in der Lage ist, seine Trinkwasserversorgung selbst zu garantieren, weil alle Oberflächengewässer vor seiner Haustür zur Kloake verkommen!“ (Deneke 1983: 81).

Das Hessische Ried beschäftigte die Darmstädter Technikphilosophie auch in der Forschung. Es fanden Kolloquiumsveranstaltungen zu dem Thema statt und Mitarbeiter*innen begannen mit Ortsbegehungen (Böhme 1984: 24). Das Ried wurde schnell zu einer Art Darmstädter Paradigma, um den Zusammenhang von Wissen, Wissenschaft, Technik und Natur in der postindustriellen Gesellschaft besser zu verstehen – ein „sozial konstruierter Naturzustand“ direkt vor der Haustür, wie es der Philosoph Böhme formulierte (Böhme 2000: 43). Das Ried bildete damit den lokalen Resonanzraum zum Projekt der „sozialen Naturwissenschaft“ (Schramm & Böhme 1985), das im Rückblick als ein gescheiterter Versuch verstanden werden kann, in Westdeutschland eine interdisziplinäre Wissenschafts- und Technikforschung aus dem Geiste der Umweltbewegung zu etablieren. Eng verbunden mit diesem Ansatz war die Hoffnung, dass sich über die Beschäftigung mit ökologisch brisanten Orten wie dem Ried die Hierarchien zwischen Fachwissenschaftler*innen, Studierenden und außeruniversitären Gegenexpert*innen verflachen würden – eine Hoffnung, die sich Ende der 1980er Jahre als Illusion herausstellte.

Das Projekt der sozialen Naturwissenschaft war zunächst ein Importprodukt vom Starnberger Max-Planck-Institut zur Erforschung der Lebensbedingungen der wissenschaftlich-technischen Welt. Hier begann sich die Gruppe „Alternativen in der Wissenschaft“ Mitte der 1970er Jahre langsam aufzulösen (Grebe 1985, Leendertz 2013). Mit der Berufung Böhmes auf die Darmstädter Professur im Jahr 1977 formierte sich die Arbeitsgruppe neu als interdisziplinärer Diskussionskreis, später als Verein. Die Naturwissenschaften könnten, so die Grundannahme, nur in Kooperation mit den Geistes- und Sozialwissenschaften eine Antwort auf die Umweltprobleme der Gegenwart finden. Erstes Studienobjekt der Arbeitsgruppe, der unter anderem Joachim Grebe, Engelbert Schramm und Wolf Schäfer angehörten – Frauen tauchen in den Publikationen aus dem Umkreis der Gruppe vermehrt erst am Ende der 1980er Jahre auf (vgl. etwa Scheich & Schultz 1989) –, war ein Wasserwerk in Ägypten. Kurz darauf geriet das Ried ins Zentrum der Aufmerksamkeit, trafen sich hier doch „alle möglichen gesellschaftlichen Akteure“ rund ums Wasser: „Bürgergruppen, Planungs- und Überwachungsbehörden, politische Instanzen, Gutachter und Gegengutachter, Wasserkonsumenten und -produzenten“ (Grebe 1985: 143–144).

Das Ried hatte aus Perspektive der Wissenschaft- und Technikforschung den Vorteil, dass die Wasserkrise ein breites Spektrum an Disziplinen und technowissenschaftlichen Expertisen anlockte. Zu den Industriewissenschaftler*innen, die an den Wasserunternehmen angestellt waren, gesellten sich in den späten 1970er Jahren Umweltwissenschaftler*innen aus dem Bereich der Hydrologie und Ingenieurswissenschaften. Sie arbeiteten häufig bei den staatlichen Behörden wie der 1971 gegründeten Hessischen Landesanstalt für Umwelt.^15^ Hinzu kamen „weiche“ Disziplinen wie die Kulturgeographie und Ethnologie, die sich angesichts des schwelenden politischen Konflikts ums Wasser für das „Regionalbewusstsein“ und regionale Identitätsbildung in den technischen Umwelten südlich von Frankfurt interessierten (Wolf & Otto 1989). Und auch Naturschützer*innen reklamierten das Ried als Naturort für sich (Sommer 1981).

Der einsetzende Konflikt über das faktische Ausmaß der Umweltkatastrophe unterminierte die Deutungsansprüche der traditionellen Expert*innen. „Es gibt“, schrieb Böhme in der Einführung zu einem seiner Forschungskolloquien, „schöne Beispiele, wo die Betroffenen selbst anfangen, Wissenschaft zu treiben, Daten zu erheben, zu forschen. Bauern im Hessischen Ried, denen von kapitalistischen Wasserfirmen der Stadt Frankfurt das Wasser abgegraben wurde, haben selbst angefangen, nachzumessen, wo eigentlich ihr Grundwasser steht“ (Böhme 1984: 152). Böhme charakterisiert den Modus von Wissenschaft, der sich im Ried beobachten ließ, als „Betroffenen-Wissenschaft“ (Böhme 1984: 151), wie sie einem häufiger in der Umwelt- und Frauenbewegung begegne. Ihr zentrales Merkmal läge darin, von konkreten gesellschaftlichen Problemen auszugehen und „Fachexpertise“ nur dann heranzuziehen, wenn sie der Problemlösung dienten. Die Zirkulation des Wissens kehre sich gewissermaßen um: „Sehr wichtig ist an diesen Ansätzen, daß ausgehend von einem bestimmten Problem die notwendigen Kompetenzen aufgesucht und nicht umgekehrt aus schon etablierten wissenschaftlichen Disziplinen die Probleme definiert werden“ (Böhme 1984: 152). Mit der Betroffenen-Wissenschaft entstünden neue Allianzen zwischen der Universität und der Gesellschaft. Eine epistemisch „entfremdete“ Gesellschaft könne sich Wissenschaft wieder „aneignen“, um den „neuen Verelendungsformen“ des „postindustriellen“ Zeitalters entgegenzuwirken (Böhme 1984: 156).

Die immensen Erwartungen, über das Studium der epistemisch-politischen Auseinandersetzungen an Orten wie dem Hessischen Ried zu einer neuen Art von Wissenschaft (und Wissenschaftsforschung) zu gelangen, wurden allerdings schnell enttäuscht. Einen nachhaltigen Dämpfer erhielt die Soziale Naturwissenschaft von der Deutschen Forschungsgemeinschaft. Zwei Projektanträge, die als Pilotstudien der Sozialen Naturwissenschaft hätten dienen sollen – eine davon zum Hessischen Ried –, erhielten eine Absage (Böhme 2000: 42–43). Die Gruppe begann sich zu zerstreuen. Viele Beteiligte zog es von den Geistes- in die Naturwissenschaften. Einige landeten bei Umweltbehörden, andere orientierten sich stärker in Richtung Ökologie (Grebe 1985). So dauerte es fast zwei Jahrzehnte, bis die erhoffte Doktorarbeit zum Hessischen Ried erschien (Kluge 2000: 87–146). Sie entstand am Institut für sozio-ökologische Forschung (ISOE) in Frankfurt, das im Jahr 1989 von Engelbert Schramm, einem tragenden Mitglied der Gruppe Soziale Naturwissenschaft, mitbegründet wurde. Auch wenn ISOE den Graben zwischen naturwissenschaftlicher und sozial- und geisteswissenschaftlicher Ökologie weiterhin überbrücken wollte – das Institut war ein gutes Beispiel dafür, dass die Fliehkräfte des Wissenschaftsbetriebs die Soziale Naturwissenschaft in Richtung der „hard sciences“ drängten.

Innerhalb der ökologischen Fachcommunity stieß das Programm der Sozialen Naturwissenschaft zudem auf starke Vorbehalte. So realisierten die Darmstädter Wissenschaftsforscher*innen schnell, dass wenige ihrer Kolleg*innen gewillt waren, im akademischen Alltag den Spagat zwischen den Natur- und Geisteswissenschaften zu vollziehen. In einem Bericht zu der im Jahr 1982 vom Bund Demokratischer Wissenschaftler (BdWi) veranstalteten Tagung „Umweltwissenschaft – Umweltpolitik“, monierte die Gruppe Soziale Naturwissenschaft, das etablierte „wissenschaftliche Selbstverständnis“ sei trotz aller Politisierung der Ökologie „ungebrochen“. Auf der Tagung „glänzten die Naturwissenschaftler“, so die Wahrnehmung, „durch ein hochgestochenes Fachvokabular und sparten nicht mit Overheadtabellen und -diagrammen, während die Sozialwissenschaftler zitatenreiche Papiere offerierten und sich in der Kenntnis ihrer Klassiker überboten“ (Anonym 1982: 59). Auch bei anderen Gelegenheiten beklagten sich Tagungsteilnehmende über die „Zahlenhuberei“ und Ignoranz der anwesenden Wissenschaftler*innen (Öko-Institut 1983): „Die Naturwissenschaftler forderten mehr (naturwissenschaftliche) Präventivkontrolle, die Sozialwissenschaft mehr (sozialwissenschaftliche) Infrastrukturplanung – alles streng über die Köpfe der Beteiligten hinweg“ (Anonym 1982: 59).

In dieser Aussage kündigte sich die Furcht vor einem Auseinanderdriften von Umweltbewegung und Umweltwissenschaft an, die sich im Laufe der 1980er Jahre verstärken sollte. In der Umweltbewegung stieg die Enttäuschung darüber, dass die wissenschaftliche Ökologie an den gesellschaftspolitischen Forderungen der Umweltbewegung wie auch an den Erkenntnissen von unorthodoxen Gegenexpert*innen nur geringes Interesse zeigte. „Das Wissen der Bewegung hat keine Auswirkungen auf das […], was ökologische Forschung macht“, beklagte sich etwa Peter Pluschke, Grünen-Politiker und späterer Umweltbeauftragte Nürnbergs, bei einer Tagung in der Evangelischen Akademie Loccum (Öko-Institut 1983: 3). Umgekehrt waren viele Wissenschaftler*innen mit den immensen gesellschafts- und wissenschaftspolitischen Erwartungen an ihr Forschungsfeld schlicht überfordert (Nelkin 1977; Bocking 2004). Die Soziale Naturwissenschaft, wie sie in Darmstadt entworfen wurde, scheiterte an den Realitäten des Wissenschaftsbetriebes, in dem – was erschwerend hinzukam – die Umweltwissenschaften im Laufe der 1980er Jahre verstärkt in Drittmittelzwang gerieten (Brämer 1982; vgl. auch Trepl 1989).

Die Forderung nach einer „alternativen“ Wissenschaft hörte man im Fachdiskurs bald nur noch selten. Am Ende des Jahrzehnts veröffentlichte Engelbert Schramm eine ernüchternde Analyse des Verhältnisses von Wissenschaft und sozialen Bewegungen. Letztere hätten sich in den Jahren zuvor stark „verwissenschaftlicht“ (Schramm 1990; vgl. auch: Kluge & Schramm 1989). Die Konsequenz der „Objektivierung“ der Konflikte war in Schramms Augen ihre schleichende Entpolitisierung. Im Grunde sei wieder das eingetreten, wogegen sich die Protestbewegungen anfangs gewehrt hätten: Expert*innen stritten sich mit Expert*innen, nur das einige von ihnen mittlerweile einen „alternativen“ Hintergrund hätten. Das Bauernopfer dieser Entwicklung – im Falle des Hessischen Rieds ist diese Formulierung durchaus wörtlich zu verstehen – war genau jener soziale Typus im Bereich des Wissens, den die Umweltbewegung in die Wissenschaft integrieren wollte: die Gegenexpert*innen, die Vermittler zwischen dem „Alltagswissen“ und der Universitätswissenschaft. Über das Ried sprach übrigens auch Schramm in seinem Aufsatz nicht mehr. Mit dem Scheitern des Projektes der Sozialen Naturwissenschaft und ähnlichen bewegungsnahen Projekten wurde es um eine historisch informierte Wissenschaft- und Technikforschung im deutschsprachigen Raum still – bis heute.^16^

## Im vergrößerten Ausschnitt

Die Umweltprobleme, die sich im Rhein-Main-Gebiet verdichteten, hatten aus wissensgeschichtlicher Perspektive einen paradoxen Nebeneffekt: „Umwelt“ wurde hier in ihrer ganzen Komplexität sichtbar. Dies bemerkte bereits im Jahr 1972 der Journalist Wolfgang Bartsch in seiner eingangs erwähnten Reportage *Umweltschutz – Menschenschutz*. Als der Journalist am Beginn des Buches seinen regionalen Zugang erläuterte, argumentierte er, sein Vorgehen entspräche ganz der neuen ökologischen Betrachtungsweise: „Daß wir einen Ausschnitt unserer Welt betrachten, wenn auch einen charakteristischen, ja einen, in dem sich Gefahren abzeichnen, die anderswo vielleicht erst später so deutlich werden wie im Rhein-Main-Gebiet, hat allein methodische Gründe: Im vergrößerten Ausschnitt erkennt man besser, was überhaupt erkennbar ist“ (Bartsch 1972: 12).

Die Geschichte der Ökologie ist reich an Regionen, die Forscher*innen halfen, im vergrößerten Ausschnitt das Große und Ganze zu erkennen: die „Äquinoktial-Gegenden“ Südamerikas, die Alexander von Humboldt in seinem berühmten *Naturgemälde der Tropenländer *darstellte; die Galapagos-Inseln, an denen Charles Darwin seine evolutionsbiologischen Hypothesen entwickelte; das Umland von Selborne, in der der Gemeindepfarrer Gilbert White am Ende des 18. Jahrhunderts seine physikotheologischen Beobachtungen zum Haushalt der Natur anstellte; oder die fiktive „Stadt im Herzen Amerikas“ (Carson 1968: 15), mit der Rachel Carson ihr Buch *Silent Spring *begann und die zu einem Leitbild für Generationen von Umweltschützer*innen werden sollte. In der „Ära der Ökologie“ (Radkau 2011) multiplizierten sich diese Regionen abermals: die Region Bitterfeld, das Ruhrgebiet, Rhein-Main, das Schweizer Mittelland, die Mailänder Agglomeration, die Industriequartiere im Norden Englands, die Bay Area, das Tennessee Valley oder das Bikini-Atoll. In und mit diesen Regionen betraten die Gegenexpert*innen die Bühne der Wissenschafts- und Technikpolitik.

Dass die Wissenschaftsgeschichte diesem neuen Typus im Bereich des Wissens wenig Aufmerksamkeit geschenkt hat, liegt vor allem daran, dass die Gegenexpert*innen nicht in die gängigen historiographischen Raster passen. Als Pfarrer war Kurt Oeser zu religiös, um als Experte durchzugehen, Jutta Ditfurth zu politisch und schlicht zu radikal, die Bauern im Hessischen Ried letztlich zu unbedeutend, um als Teil der Geschichte dieses Forschungsfeldes gelten zu können. Oft unterlaufen die Gegenexpert*innen mit ihrem regionalen Aktionsradius zugleich die gängigen geographischen „scales“ (vgl. Coen 2018; Westermann & Höhler 2020) der Geschichtsschreibung.

Im Umfeld der Anthropozändebatte haben sich Historiker*innen in den letzten Jahren daran gewöhnt, die Geschichte von Umwelt entweder im ganz Kleinen – dem situierten Wissen einzelner Akteure – oder im ganz Großen – dem globalen Klima – zu suchen. Interessanterweise waren viele Umweltwissenschaftler*innen genau in einem Bereich dazwischen aktiv, nämlich inmitten der technischen Umwelten des 20. Jahrhunderts. Sie bündelten lokales Wissen und verbanden es mit den „ganz großen“ Fragen. Die Geschichte der ökologischen Gegenexpert*innen lädt insofern dazu ein, dem Diskurs um das Anthropozän einen weiteren, „nahräumigeren“ *scale *hinzuzufügen und ihn damit stärker zu provinzialisieren: den *meso scale *(Güttler 2019). Die schmutzigen und lärmenden technischen Umwelten der „Ära der Ökologie“ können also ein guter Ausgangspunkt sein, um im vergrößerten Ausschnitt die Rolle von Wissen und Wissenschaft in den Umweltkrisen des 20. und 21. Jahrhunderts neu denken – als Teil einer politischen Wissensgeschichte.

## Dank

Dieser Text entstand an der Professur für Wissenschaftsforschung der ETH Zürich. Ich danke den Mitarbeiter*innen der Professur und den Teilnehmer*innen des Workshops „Scientific Political Activism“ am Zentrum für Geschichte des Wissens, Zürich, im Mai 2019. Für wertvolle Rückmeldungen und Hinweise zu diesem Text gilt mein besonderer Dank: Niki Rhyner sowie Anna-Maria Schmidt, Alexander von Schwerin, Max Stadler und den beiden anonymen Gutachter*innen.

## Footnotes

1 Als weitere Problemfälle nannte Korda die Saar, die Räume Salzgitter, Hamburg, Stuttgart und Ingolstadt.

2 Eindrücklichstes Beispiel hierfür ist die weiterhin wichtigste Studie zur „Naturpolitik“ in Westdeutschland, die den Gegenexpert*innen gerade mal drei Seiten widmet: Engels (2006): 202–205. Eine Ausnahme aus der neuen Literatur zur Anti-Atomkraft-Bewegung bildet: Augustine (2018): 74–92.

3 Vgl. zum Begriff des situierten Wissens: Haraway (1988).

4 Zur Geschichte der Interdisziplinarität in der BRD vgl. Schregel (2016).

5 In den bundesdeutschen Organen wurde außerdem wiederholt über die wissenschaftlichen und medizinischen Studien zur Auswirkung des Lärms auf die Umgebung von Zürich-Kloten und Paris-Orly berichtet.

6 Zur Vorgeschichte der Diskussionen innerhalb der Anti-Fluglärm-Bewegung siehe: Martin (1969).

7 Vgl. zur Diskussion rund um die „Objektivität“ der Lärmmessung und die Einführung von Lärmquotienten: Bijsterveld (2008): 193–232. Eine Geschichte der Lärmforschung im deutschsprachigen Raum liegt bislang nicht vor. Einen guten Überblick über die Fachdiskussion bis in die frühen 1980er Jahre findet sich in: Institut für Stadtgeschichte, Frankfurt; Kommission zur Abwehr des Fluglärms; Nr. 4.

8 Ein gutes Beispiel ist die Reaktion von Wissenschaftler*innen auf die Veröffentlichung der DFG-Fluglärmstudien in den Medien, etwa: Anonym (1975).

9 Ein ausführlicher Bericht Oesers über die Tagung findet sich in: Institut für Stadtgeschichte, Frankfurt; Kommission zur Abwehr des Fluglärms, Nr. 7 (34. Sitzung, 25. März 1971).

10 Vgl. Oesers Vortrag „Lärmbelästigung“ auf dem Hessischen Naturschutztag 1970, in: Institut für Stadtgeschichte, Frankfurt; Kommission zur Abwehr des Fluglärms, Nr. 1.

11 Einen guten Einblick in die verschiedenen beteiligten Disziplinen liefert neben dem Protokoll und dem Gutachten des Hearings zur Startbahn-West im Hessischen Landtag außerdem: Hessischer Minister für Landesentwicklung, Umwelt, Landwirtschaft und Forsten (1981).

12 Frankfurter Initiative Volksbegehren/Volksentscheid „Keine Startbahn West, Rundbrief Nr. 4, Frankfurt“ vom 4. August 1981. Archiv für soziale Bewegungen, Freiburg, 12.2.7.6 II: Startbahn West (Frankfurt), grauer Ordner.

13 Diese Messungen wurden später von drei von der Landesregierung beauftragten Forschungsinstituten nicht bestätigt: Anonym (1981c).

14 Vgl. zur Sozialgeschichte des linksalternativen Lebens in der BRD: Reichardt (2014).

15 Vgl. hier insbesondere die Reihe „Umweltplanung und Umweltschutz“, die vom Hessischen Landesamt für Umwelt herausgeben wurde, erschien beispielweise bei der Hessischen Landesanstalt für Umwelt (1985).

16 Forscher*innen wie Helga Nowotny, die im Sommersemester 1980 im Darmstädter Kolloquium mit einem Vortrag zum „Verhalten wissenschaftlicher Experten in der öffentlichen Diskussion um die Kernenergie (am Beispiel Österreich)“ vertreten war, zog es verstärkt in die Soziologie und in die Wissenschaftspolitik. Andere, wie Karin Knorr-Cetina, hatten sich zu diesem Zeitpunkt längst in die angloamerikanischen *Science and Technology Studies* orientiert, wo es für die – auch in Darmstadt angestrebten – Verbindungen zwischen Soziologie, Naturwissenschaften und Wissenschaftsgeschichte einen größeren Resonanzraum gab. Die Schnittstelle von Wissenschaftsforschung und -geschichte, aus der die *history of science *in Großbritannien und den USA bis in die 2000er Jahre hinein einen erheblichen Teil ihrer intellektuellen Energie zog (Jasanoff 2000; Daston 2009), konnte sich im deutschsprachigen Raum nur situativ herausbilden.
